# Mesenchymal stem cells alleviate experimental rheumatoid arthritis through microRNA-regulated IκB expression

**DOI:** 10.1038/srep28915

**Published:** 2016-06-29

**Authors:** Xin Yan, Yurong Cen, Qin Wang

**Affiliations:** 1 Department of Rheumatology, Shanxi University affiliated the First Hospital, Taiyuan 030001, China; 2 Department of Nephrology and Rheumatology, Shanghai Jiaotong University affiliated Sixth People’s Hospital, South Campus, Shanghai 201400, China; 3 Department of Nephrology and Rheumatology, Nanfang Medical University affiliated Fengxian Hospital, South Campus, Shanghai 201400, China

## Abstract

Previous studies have demonstrated that mesenchymal stem cell (MSC) transplantation reduces the severity of collagen-induced arthritis (CIA) in mice, which is a model for rheumatoid arthritis (RA) in humans. However, the underlying molecular mechanisms remain ill-defined. Here, we showed that MSC transplantation reduced the activities of NF-κB signaling and decreased microRNA-548e (miR-548e) levels in the joint tissue in CIA-mice, seemingly through activation of transforming growth factor β receptor signaling. Bioinformatics analyses revealed that miR-548e inhibited protein translation of the NF-κB inhibitor, IκB, through binding to the 3′-UTR of the IκB mRNA. MSCs co-transplanted with adeno-associated virus (AAV) carrying miR-548e abolished the therapeutic effects of MSCs on CIA. On the other hand, transplantation of AAV carrying antisense of miR-548e (as-miR-548e) partially mimicked the effects of MSC transplantation on CIA. Together, these data suggest that MSC transplantation may alleviate experimental RA partially through suppressing miR-548e-mediated IκB inhibition.

Rheumatoid arthritis (RA) is a chronic inflammatory disease that primarily affects the joints, causing articular destruction and associated pain, stiffness, and synovitis^1^,^2^,^3^. In addition to causing a perturbation of both the innate and adaptive immune systems^1^,^2^,^3^,^4^, RA has been associated with the presence of serum autoantibodies against self-proteins and rheumatoid factors^5^,^6^,^7^,^8^. However, the exact triggers of this RA phenotype remain unknown. Hence, the development of relevant animal models of RA in humans appears to be crucial for understanding the molecular mechanisms underlying the pathogenesis of RA.

Collagen-induced arthritis (CIA) shares many similarities with human RA^9^,^10^,^11^,^12^,^13^. CIA was first applied in rodents, including rats and mice^14^,^15^. The susceptibility of developing CIA depends on the animal strains. DBA/1J mice are most widely used in the CIA model^16^,^17^,^18^,^19^. Clinical signs similar to human RA typically develop in DBA/1J mice 21–25 days after the initial inoculation, and have been associated with both B- and T-lymphocyte responses with the production of anti-collagen type II antibodies and collagen-specific T cells^16^,^17^,^18^,^19^. Disease severity is expected to peak at approximately day 35, after which DBA/1J mice undergo remission, marked by increased concentrations of serum IL-10 and transforming growth factor β (TGFβ) and a subsequent decrease in pro-inflammatory cytokines: interleukin (IL)-1β, tumor necrosis factor (TNF)-α and IL-6^20^,^21^,^22^.

Nuclear factor-κB (NF-κB) has been well recognized as a pivotal regulator of inflammation in RA^23^,^24^,^25^. However, recent experiments have shown a broad involvement of NF-κB in other aspects of RA pathology, including development of T helper 1 responses, aberrant apoptosis and proliferation of RA-associated fibroblast-like synovial cells^26^. NF-κB is a group of dimeric transcription factors comprised of the Rel family of proteins that include RelA (p65), c-Rel, RelB, NF-κB1 (p50), and NF-κB2 (p52)^23^,^24^,^25^. The most abundant form in activated cells is the RelA/NF-κB1 (p65/p50) heterodimer^23^,^24^,^25^. NF-κB resides in the cytoplasm in its latent form, but translocates to the nucleus upon stimulation^23^,^24^,^25^. The cytoplasmic retention of NF-κB results from its interaction with inhibitory proteins known as IκB^23^,^24^,^25^. Insufficient IκB results in the detachment of NF-κB from IκB, and the detached NF-κB subsequently enters the nucleus to initiate gene transcription^23^,^24^,^25^. Of note, rodent studies have used specific inhibitors of the NF-κB pathway to treat RA and have achieved promising results^23^,^24^,^25^.

Mesenchymal stem cells (MSCs) are multipotent progenitor cells that can differentiate into tissues of mesenchymal lineage, including bone, cartilage and adipose tissue^27^,^28^,^29^. Several studies have reported therapeutic effects of allogenic or xenogenic MSC transplantation in CIA mice^30^,^31^,^32^,^33^,^34^,^35^,^36^. However, the underlying molecular basis of these effects is not fully understood.

Here, we showed that MSC transplantation reduced the activity of NF-κB signaling and decreased microRNA-548e (miR-548e) levels in the joint tissue in CIA-mice, seemingly through activation of transforming growth factor β receptor signaling. Bioinformatics analyses revealed that miR-548e inhibited protein translation of the NF-κB inhibitor, IκB, through binding to the 3′-UTR of the IκB mRNA. MSCs co-transplanted with adeno-associated virus (AAV) carrying miR-548e abolished the therapeutic effects of MSCs on CIA. On the other hand, transplantation of AAV carrying antisense of miR-548e (as-miR-548e) partially mimicked the effects of MSC transplantation on CIA. Together, these data suggest that MSC transplantation may alleviate experimental RA, partially through suppressing miR-548e-mediated IκB inhibition.

## Materials and Methods

### Protocol approval

All the experimental methods in the current study have been approved by the research committee at Medical College of Shanghai Jiao Tong University. All the experiments have been carried out in accordance with the guidelines from the research committee at Medical College of Shanghai Jiao Tong University. All mouse experiments were approved by the Institutional Animal Care and Use Committee at Shanghai Jiao Tong University (Animal Welfare Assurance). Surgeries were performed in accordance with the Principles of Laboratory Care, supervised by a qualified veterinarian.

### Isolation, culture and differentiation of MSCs

Bone-marrow derived MSCs were isolated from male DBA/1J mice (Shanghai Laboratory Animal Center, China) at 8 weeks of age. MSCs were collected from femurs and tibias by flushing with DMEM culture medium (Dulbecco’s Modified Eagle’s Medium, Gibco, San Diego, CA, USA). The cells were centrifuged and re-suspended in DMEM containing inactivated 10% fetal bovine serum (FBS, Gibco), 3.7 g/l HEPES (N-2-hydroxyethylpiperazine-N’-2-ethane-sulphonic acid, Sigma-Aldrich, St. Louis, MO, USA), 1% 200 mmol/l L-glutamine 100× (Gibco) and 1% PSA (Gibco). The cell number and viability were determined by trypan blue staining (Gibco). The cells were incubated in a humidified chamber with 5% CO_2_ at 37 °C for 72 h. The adherent cells were considered MSCs and were maintained in culture until reaching 80% confluence. The MSCs were then washed, incubated with trypsin-ethylenediaminetetraacetic acid (EDTA) (StemCell Technologies, Vancouver, Canada) and prepared to be frozen with a solution containing 10% dimethyl sulfoxide (DMSO, MP Biomedicals, Santa Ana, USA) in culture medium. After confirmation of MSC properties, a positive clone was selected by chondrogenetic, osteogenic, and adipogenic differentiation assays. For chondrogenetic induction, 2.5 × 10^5^ MSCs were induced with 5 ml chondrogenetic induction medium containing 10 μg transforming growth factor β1 (TGFβ1, R&D System, Los Angeles, CA, USA), 50 μg insulin growth factor 1 (IGF-1, R&D System). The cells were maintained in the chondrogenetic induction medium for 14 days and then subjected to Alcian blue staining. For osteogenic induction, cells were digested and seeded onto a 24-well plate at a density of 10^4 ^cells/well, and then maintained in osteogenic induction medium containing 10 nmol/l Vitamin D3 (Sigma-Aldrich) and 10 mmol/l β-phosphoglycerol and 0.1 μmol/l DMSO for 14 days before subjected to Von kossa staining. For adipogenic induction, cells were digested and seeded onto a 24-well plate at a density of 10^4 ^cells/well, and then maintained in the adipogenic induction medium containing 0.5 mmol/l 3-isobutyl-1-methylxanthine (IBMX), 200 μmol/l indomethacin, 10 μmol/l insulin and 1 μmol/l DMSO for 14 days before subjected to Oil red O staining.

### Preparation of AAV-miR-548e, AAV-as-miR548e and AAV-null

The Human Embryonic Kidney 293 cell line (HEK293) was used for virus production. We used a pAAV-CMVp-GFP plasmid (Clontech, Mountain View, CA, USA), a packaging plasmid carrying the serotype 8 rep and cap genes, and a helper plasmid carrying the adenovirus helper functions (Applied Viromics, LLC. Fremont, CA, USA) for generating AAVs in this study. The sequence for the miR-548e construct is 5′-AAAAACUGAGACUACUUUUGCA-3′. The sequence for the as-miR-548e construct is 5′-UGCAAAAGUAGUCUCAGUUUUU-3′. These constructs with a 2A sequence were cloned into a pAAV-CMVp-GFP backbone at the site between CMVp and GFP. AAVs were produced by co-transfecting HEK293 cells with the prepared pAAV-CMVp-miR-548e/pAAV-CMVp-as-miR-548e/pAAV-CMVp-Null plasmids, R2C8 (containing AAV2 Rep and AAV8 capsid genes) and plAd5 (containing adenovirus helper genes) by Lipofectamine 2000 (Invitrogen, St. Louis, MO, USA). The viruses were purified using CsCl density centrifugation and then titered by a quantitative densitometric dot-blot assay.

### Mouse CIA model

Treatment was initiated after the onset of disease, when arthritis had become well established approximately 3 weeks after the primary immunization. Clinical assessment was continued during the subsequent 4 weeks. Male DBA/1J mice (Shanghai Laboratory Animal Center, China) at 8 weeks of age were injected intradermally at the base of the tail with 200 μg bovine type II collagen (CII; Chondrex, Redmond, WA, USA) emulsified in Freund’s complete adjuvant (1:1, v/v; Chondrex) containing 200 μg Mycobacterium tuberculosis H37Ra (Chondrex). Two weeks later the mice were given intradermal booster injections of 100 μg CII in incomplete Freund’s adjuvant (1:1, v/v; Chondrex). Mice were monitored for signs of arthritis based on paw swelling and clinical arthritis scores. Paw thickness was measured with 0~10 mm calipers (Kroeplin, Schluchtern, Germany). For the clinical score, a 4-point scale was used, where 0 = normal, 1 = slight swelling and erythema, 2 = pronounced edema, and 3 = joint rigidity, as has been described for classic CIA^37^,^38^. Each limb was graded, and the mean was taken for each animal. Clinical arthritis scoring was performed by two observers independently. Mice were randomly distributed to each treatment group (n = 5) at 1 week after 2nd booster injection of CII when arthritis scores were between 1 and 1.5. Mice with a clinical arthritis score below 1 were excluded from the experiment to minimize the variance of arthritis score between groups.

### Transplantation of MSCs and AAV injection

CIA mice were intraperitoneally injected with 100 μl phosphate-buffered saline (PBS control, n = 5; Invitrogen, Carlsbad, CA, USA) or 100 μl PBS containing 10^6^ MSCs (n = 5 per each group). AAVs (10^9^ AAV-miR-548e or AAV-as-miR-548e viral particles in 100 μl PBS; control: same amount of AAV-null virus in 100 μl PBS) was injected intraperitoneally at together with MSCs (AAV-miR-548e) or alone (AAV-as-miR-548e) (n = 5 per each group). All mice were sacrificed 4 weeks after the MSC transplantation. The sera and limbs of all animals were collected for analysis.

### Histological assessments of CIA

At 4 weeks after MSC/AAV injection, mice were anesthetized and euthanized for analysis. Formalin (10%, Merck & Co. Inc, NY, USA)-fixed limbs were decalcified in EDTA for 4 weeks and embedded in paraffin (Merck & Co. Inc) using standard histologic techniques. Serial 4-μm sections were cut and stained with hematoxylin and eosin (Sigma-Aldrich) to examine morphologic features and determine the histological arthritis score. Sections were evaluated histopathologically and scored for synovial inflammation and bone erosion, according to published criteria^39^. Briefly, for inflammation score 0: No inflammation; score 1: Slight thickening of lining layer or some infiltrating cells in sublining layer; score 2: Slight thickening of lining layer plus some infiltrating cells in sublining layer; score 3: Thickening of lining layer, influx of cells in sublining layer and presence of cells in the synovial space and score 4: Synovium highly infiltrated with many inflammatory cells. For cartilage erosion score 0: No destruction; score 1: Minimal erosion limited to single spots; score 2: Slight to moderate erosion in a limited area; score 3: More extended erosions and score 4: General destruction.

### Measurement of inflammatory cytokines

Serum levels of the inflammatory cytokines murine IL-1β, IL-6, interferon (IFN)-γ, TNF-α, and IL-10 were determined using the Luminex multiplex cytokine assay (Luminex 200 system, Millipore, Billerica, MA, USA) according to the manufacturer’s recommendations. The level of TGFβ1 was measured using a commercially available mouse TGFβ1 enzyme-linked immunosorbent assay (ELISA) kit (R&D Systems).

### Quantitative real-time PCR (RT-qPCR)

MiRNA and total RNA were extracted from cultured cells with miRNeasy mini kit or RNeasy kit (Qiagen, Hilden, Germany), respectively, for cDNA synthesis. Quantitative real-time PCR (RT-qPCR) was performed in duplicates with QuantiTect SYBR Green PCR Kit (Qiagen). All primers were purchased from Qiagen. Data were collected and analyzed with the Rotorgene software accompanying the PCR machine, using 2−△△Ct method for quantification of the relative mRNA expression levels. Values of genes were first normalized against α-tubulin, and then compared to controls.

### Western blot

Nuclear and cytoplasmic proteins were isolated with Nuclear and Cytoplasmic Extraction Kit (Thermo Scientific, Rockford, IL, USA) from the joint tissue of the mice. Primary antibodies were rabbit anti-NF-κB (p65; detection of total protein; Cell Signaling, San Jose, CA, USA; Catalog number: 8242 s; diluted 1:1000), rabbit anti-IκB (Cell Signaling; Catalog number: 4812 s; diluted 1:1000), anti-LaminB1 (Cell Signaling; Catalog number: 12586 s; diluted 1:1000) and anti-α-tubulin (Cell Signaling; Catalog number: 2125 s; diluted 1:1000). Secondary antibody is HRP-conjugated anti-rabbit (Dako, Carpinteria, CA, USA; Catalog number: P0448; diluted 1:1000). Figure images were representative from 5 repeats. LaminB1 was used as a protein loading control for nuclear protein, and α-tubulin was used a protein loading control for cytoplasmic protein.

### Isolation and culture of synovial fibroblasts

For isolation of human synovial fibroblasts, the healthy joint tissue from a deceased 55-year-old male was dissected and placed in separate large petri dishes. For isolation of mouse synovial fibroblasts, the joint tissue was obtained from male DBA/1J mice at 8 weeks of age. A scalpel was used to cut tissue pieces as small as possible. Thereafter, the tissue pieces were carried over to a new 10 ml tube, and 2.5 ml 30 mg/ml collagenase (Sigma-Aldrich) was added to the tissue to incubate for 60 min at 37 °C. In the last 10 minutes of this 60 minute incubation, 5 ml 0.25% trypsin (Sigma-Aldrich) was added to the tube. Afterwards, the tube was centrifuged for 5 minutes at 300 g to pellet the cells. The cells were then re-suspended and seeded into a six-well plate that was pre-coated with a 0.1% gelatin solution for 20 minutes at 37 °C. The gelatin was removed from the dish before the cells were seeded. Passaging of the cells was performed when confluence of the culture plate was achieved. The culture media for human or mouse synovial fibroblasts consisted of DMEM (Gibco) supplemented with 15% FBS. Transfection of the synovial fibroblasts was performed by Lipofectamine 2000 reagent (Invitrogen, Carlsbad, CA, USA) for 24 hours.

### Luciferase-reporter activity assay

Luciferase-reporters were successfully constructed using molecular cloning technology. The target sequence was inserted into a pGL3-Basic vector (Promega, Madison, WI, USA) to obtain pGL3-IκB-3′ UTR, which contained the miR-548e binding sequence (IκB-3′ UTR sequence). miR-548e-modified fibroblasts were seeded in 24-well plates for 24 hours, after which they were transfected with 1 μg of Luciferase-reporter plasmids per well using PEI Transfection Reagent. Then luciferase activity was measured using the dual-luciferase reporter gene assay kit (Promega), according to the manufacturer’s instructions.

### Statistical analysis

All values represent the mean ± standard deviation (SD). Statistical analysis of group differences was carried out using a one-way analysis of variance (ANOVA) test (SPSS 12.0, Chicago, IL, USA) followed by the Fisher’s Exact Test to compare two groups. Ten mice were used in each experimental group. A value of p < 0.05 was considered statistically significant after Bonferroni correction.

## Results

### Preparation of mouse MSCs

**Figure 1:**
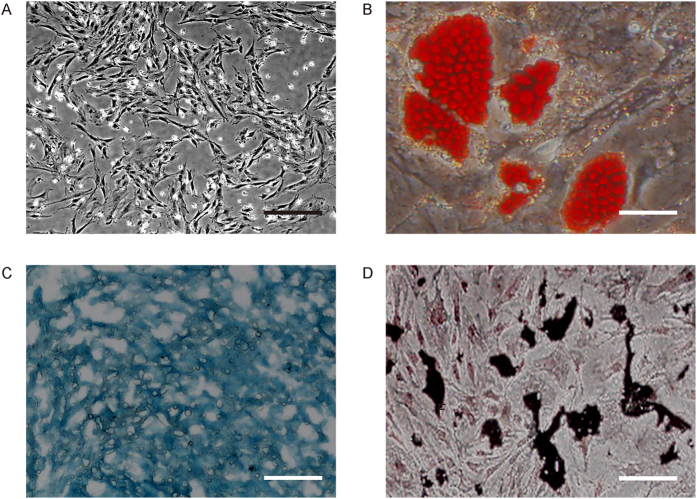
Preparation of mouse MSCs. (**A**) Mouse bone marrow derived MSCs in culture. (**B**–**D**) A selected clone of MSCs was examined for differentiation properties by Von kossa staining to evaluate osteogenic induction (**B**), Oil red O staining to evaluate adipogenic induction (**C**) and Alcian blue staining to evaluate chondrogenetic induction (**D**). Scale bars are 50 μm.

We studied the molecular mechanisms underlying the therapeutic effects of MSCs on CIA. For this aim, we isolated mouse MSCs from bone marrow (Fig. 1A), and confirmed the MSC properties of a selected clone by differentiation assay (Fig. 1B–D).

### Therapeutic effects of MSCs in CIA mice

**Figure 2:**
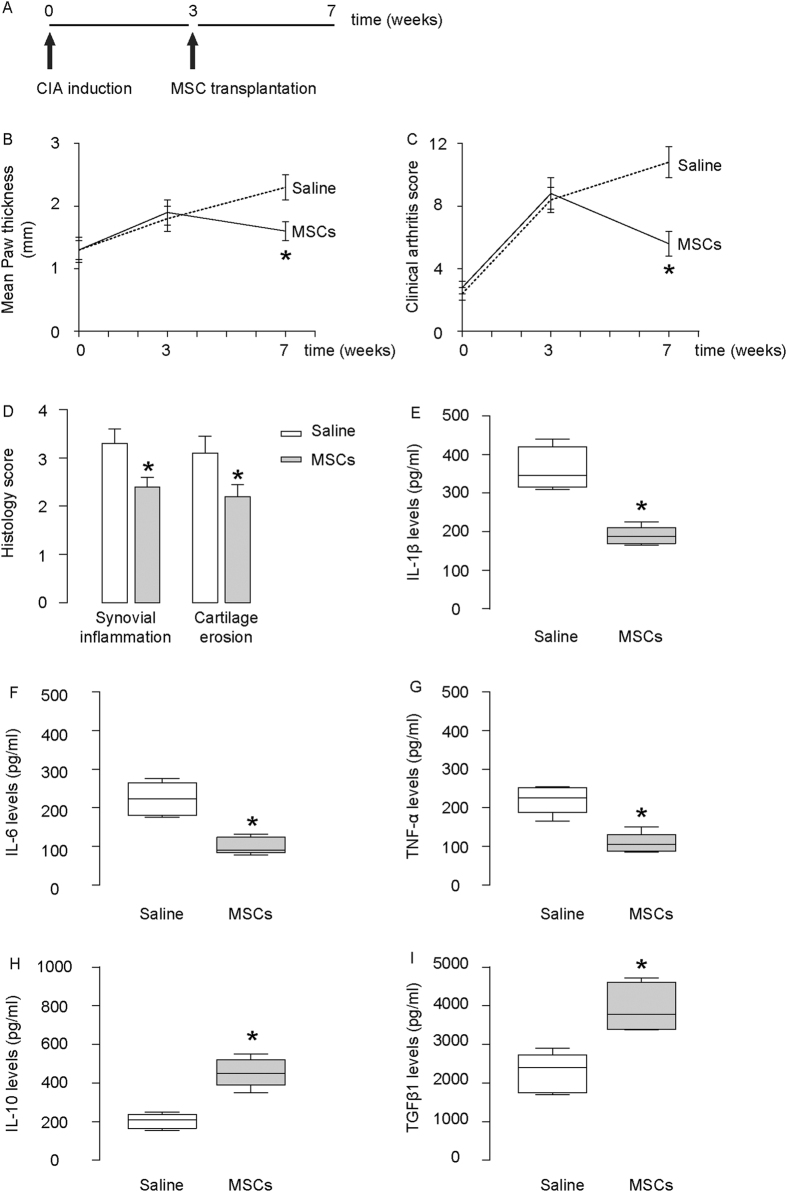
Therapeutic effects of MSCs in CIA mice. (**A**) A schematic to show the experiments for evaluation of the effects of MSCs on CIA. Saline: CIA induced, transplanted with saline. MSCs: CIA induced, transplanted with MSCs. (**B**) Paw thickness. (**C**) Clinical arthritis score for all limps. (**D**) Histological arthritis score. (**D**) ELISA for IL-1β, IL-6, TNF-α, IL-10 and TGFβ1 levels in inflamed joints of CIA mice, treated with MSCs (MSCs), or without MSCs (saline). *p < 0.05. N = 5.

The experiments to evaluate the effects of MSCs on CIA are shown in a schematic (Fig. 2A). We found that MSC transplantation significantly attenuated the severity of arthritis, based on analyses of paw thickness (Fig. 2B), clinical arthritis score (Fig. 2C), and histological arthritis score (Fig. 2D). Moreover, MSC transplantation reduced protein levels of IL-1β (Fig. 2E), IL-6 (Fig. 2F) and TNF-α (Fig. 2G), and increased levels of IL-10 (Fig. 2H) and TGFβ1 (Fig. 2I) in the inflamed joints of CIA mice. These data confirm that MSC transplantation has therapeutic effects on CIA in mice.

### MSC reduces CIA-induced increases in NF-κB activities in synovial fibroblasts

**Figure 3:**
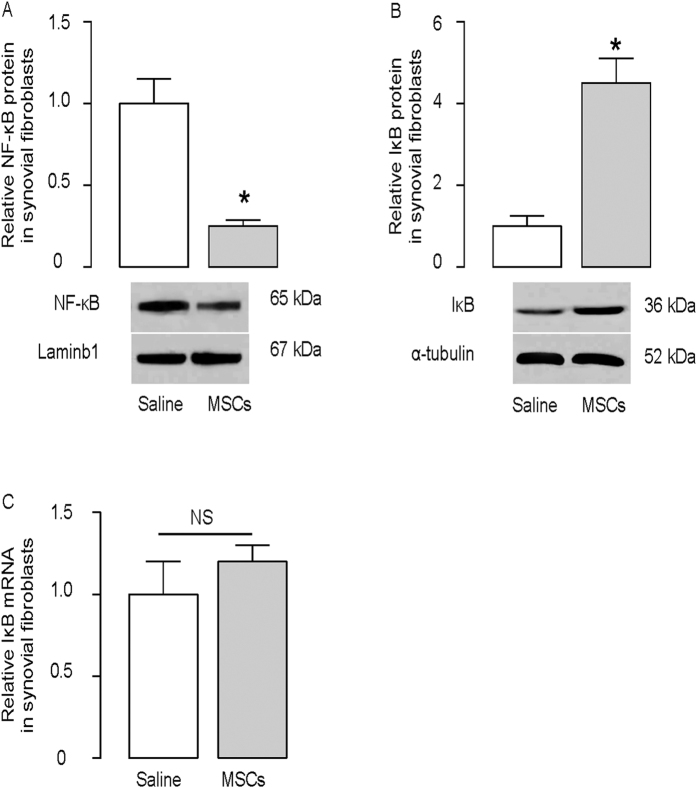
MSC reduces CIA-associated increases in NF-κB activities in synovial fibroblasts. (**A**,**B**) The nuclear NF-κB (p65) levels (**A**) and cytoplasmic IκB levels (**B**) in synovial fibroblasts in CIA-mice treated with MSCs (MSCs), or without MSCs (saline). (**C**) The mRNA levels of IκB in synovial fibroblasts. *p < 0.05. NS: non-significant. N = 5.

Synovial fibroblasts play a pivotal role in the development of CIA. Thus, in order to examine changes in NF-κB signaling by MSC transplantation in CIA-mice, we analyzed the levels of NF-κB and IκB proteins in synovial fibroblasts isolated from mouse joints. We found that MSC transplantation decreased nuclear NF-κB protein (p65, Fig. 3A), and increased cytoplasmic IκB protein (Fig. 3B) in synovial fibroblasts. However, the mRNA levels of IκB in synovial fibroblasts were not altered by MSC transplantation (Fig. 3C). These data suggest the possibility of post-transcriptional control of IκB by MSC transplantation.

### MiR-548e targets 3′-UTR of IκB mRNA to inhibit its translation in synovial fibroblasts

**Figure 4:**
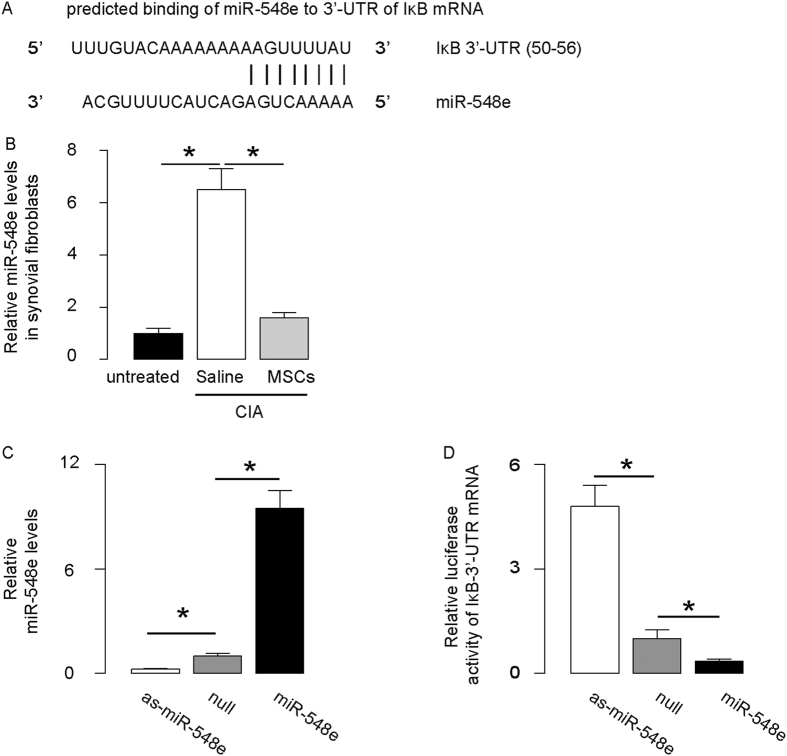
MiR-548e targets 3′-UTR of IκB mRNA to inhibit its expression in synovial fibroblasts. (**A**) Bioinformatics analyses of IκB target sequence, showing that miR-548e binds to 3′-UTR of IκB mRNA at 50^th^–56^th^ base site. (**B**) The levels of miR-548e in synovial fibroblasts significantly increased after CIA (compared to untreated), but then significantly reduced after MSC transplantation (MSCs). (**C**) Human synovial fibroblasts were transfected with either miR-548e or antisense for miR-548e (as-miR-548e). The synovial fibroblasts were also transfected with a null sequence as a control (null). Modulation of miR-548e levels in synovial fibroblasts was confirmed by RT-qPCR. (**D**) The miR-548e-modified synovial fibroblasts were transfected with 1 μg of IκB-3′ UTR Luciferase-reporter plasmid and examined for luciferase activities. *p < 0.05. N = 5.

Next, we performed bioinformatics analyses to identify the IκB target sequence for miRNAs that bind to the 3′-UTR of IκB mRNA. From these candidates, we specifically found that miR-548e bound to 3′-UTR of IκB mRNA at 50^th^–56^th^ base site (Fig. 4A), and the levels of miR-548e in synovial fibroblasts were significantly increased after CIA (compared to the untreated), but significantly reduced by MSC transplantation (Fig. 4B). To determine whether the binding of miR-548e to IκB mRNA is functional, we isolated synovial fibroblasts from a healthy human, and transfected the cells with either miR-548e or antisense for miR-548e (as-miR-548e). The synovial fibroblasts were also transfected with a null sequence as a control (null). Modulation of miR-548e levels in these cells was confirmed by RT-qPCR (Fig. 4C). Then, these miR-548e-modified cells were transfected with 1 μg IκB-3′-UTR Luciferase-reporter plasmid. We found that the luciferase activities in miR-548e-depleted cells were significantly higher than the control, while the luciferase activities in miR-548e-overexpressing cells were significantly lower than the control (Fig. 4D). These data suggest that miR-548e targets 3′-UTR of IκB mRNA to inhibit its protein translation.

### MSCs may suppress miR-548e in synovial fibroblasts through TGFβ receptor signaling

**Figure 5:**
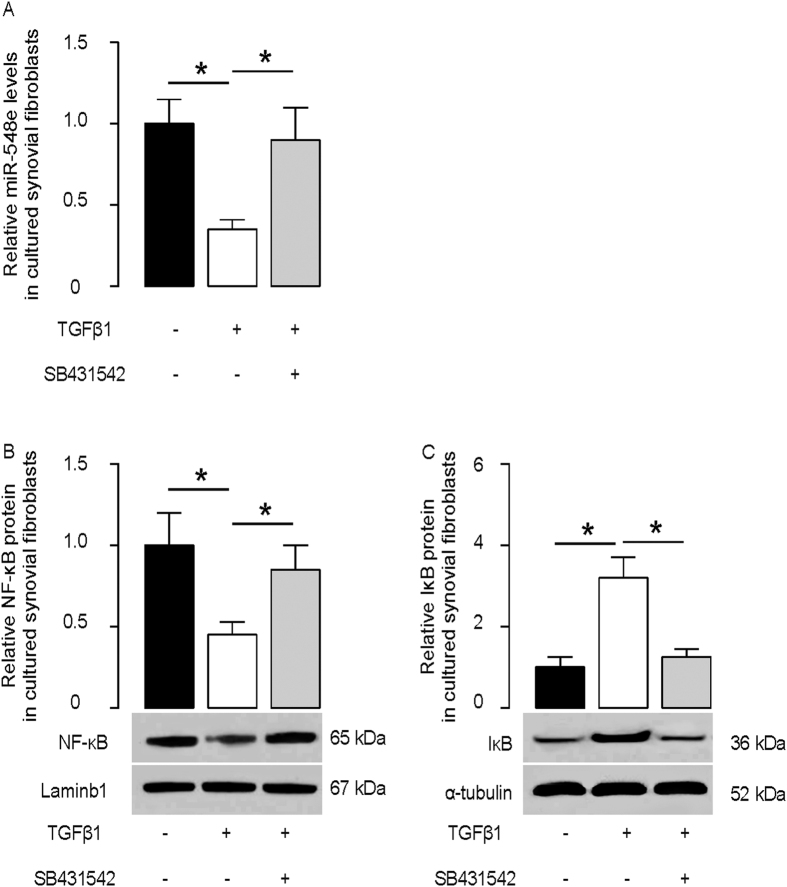
MSCs suppress miR-548e in synovial fibroblasts through TGFβ receptor signaling. We gave cultured mouse synovial fibroblasts 10 μmol/l TGFβ1 with or without a TGFβ receptor inhibitor SB431542 (10 μmol/l). (**A**) TGFβ1 decreased miR-548e levels in synovial fibroblasts, which was abolished by SB431542. (**B**,**C**) TGFβ1 decreased nuclear NF-κB protein (**B**) and increased cytoplasmic IκB protein (**C**), which were also abolished by SB431542. *p < 0.05. N = 5.

Since TGFβ1 is a well-known growth factor that is produced and secreted by MSCs, and since TGFβ1 levels are significantly increased in CIA-mouse joints after MSC transplantation, we hypothesized that MSCs may suppress miR-548e levels in synovial fibroblasts through TGFβ receptor signaling. To test this hypothesis, we gave cultured mouse synovial fibroblasts 10 μmol/l TGFβ1 with or without a TGFβ receptor inhibitor SB431542 (10 μmol/l). SB431542 inhibits TGFβ receptor signaling through suppression of TGFβ receptor 1 phosphorylation. We found that TGFβ1 decreased miR-548e levels in synovial fibroblasts, an effect which was abolished by SB431542 (Fig. 5A). Moreover, TGFβ1 increased cytoplasmic IκB protein levels and decreased nuclear NF-κB protein levels, effects which were also abolished by SB431542 (Fig. 5B,C). These data suggest that MSCs may suppress miR-548e in synovial fibroblasts through TGFβ receptor signaling.

### Overexpression of miR-548e abolishes the therapeutic effects of MSCs on CIA

**Figure 6:**
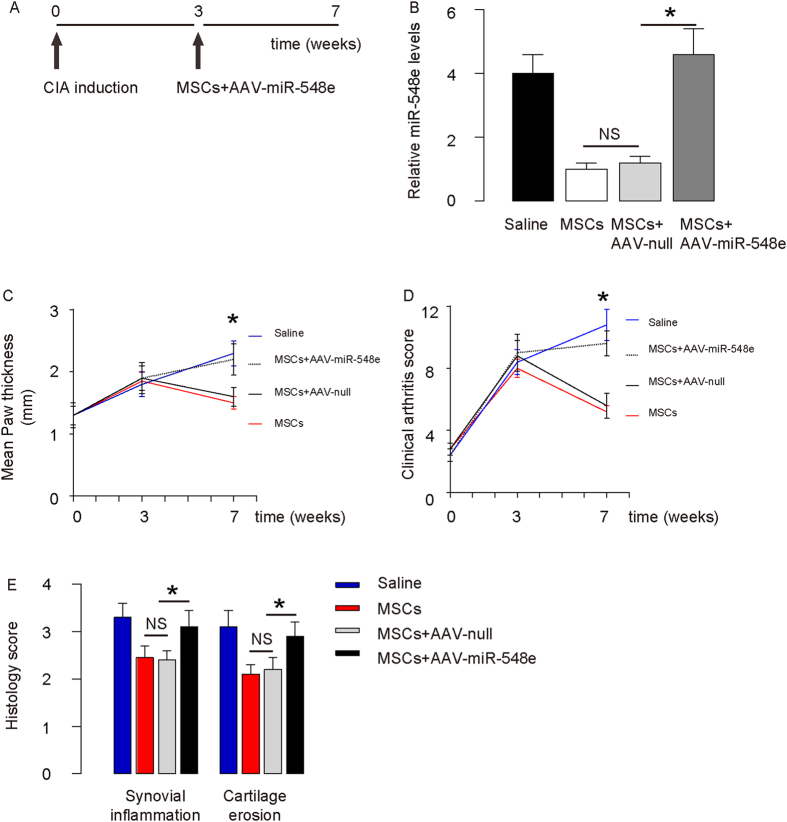
Overexpression of miR-548e abolishes the therapeutic effects of MSCs on CIA. (**A**) We co-transplanted AAV-miR-548e viruses with MSCs, and examined the effects on MSC transplantation in CIA-mice. (**B**) RT-qPCR on articular miR-548e. (**C**–**F**) Overexpression of miR-548e abolished the therapeutic effects of MSCs on CIA, by paw thickness (**C**), clinical arthritis score for all limps (**D**) and histological arthritis score (**E**). *p < 0.05. NS: non-significant. N = 5.

Next, we co-transplanted AAV-miR-548e with MSCs and examined the effects in CIA-mice (Fig. 6A). First, the effects of AAV-miR-548e on miR-548e levels in synovial fibroblasts were confirmed (Fig. 6B). We found that overexpression of miR-548e in synovial fibroblasts abolished the therapeutic effects of MSCs on CIA, based on analyses of paw thickness (Fig. 6C), clinical arthritis score (Fig. 6D) and histological arthritis score (Fig. 6E).

### Inhibition of miR-548e alone mimics the therapeutic effects of MSCs on CIA

**Figure 7:**
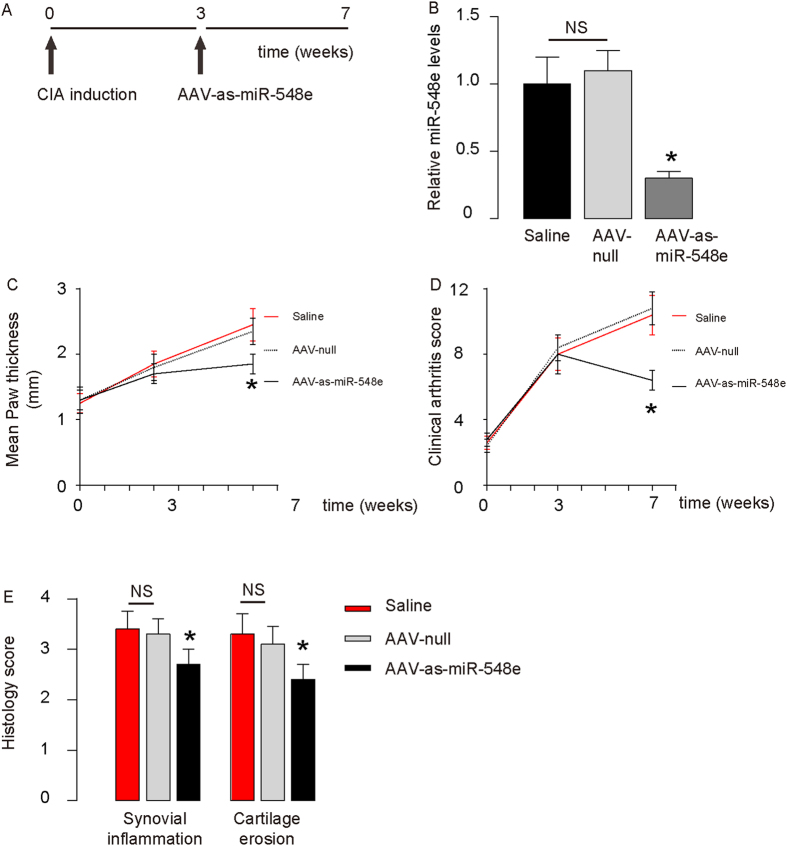
Inhibition of miR-548e alone mimics the therapeutic effects of MSCs on CIA. (**A**) We injected AAV-as-miR-548e viruses into CIA-mice, and examined the effects on CIA severity. (**B**) RT-qPCR on articular miR-548e. (**C**–**F**) Expression of as-miR-548e alone, without need of MSC transplantation, mimicked the therapeutic effects of MSCs on CIA, by paw thickness (**C**), clinical arthritis score for all limps (**D**) and histological arthritis score (**E**) *p < 0.05. NS: non-significant. N = 5.

Finally, we injected AAV-as-miR-548e viruses into CIA-mice and examined the effects on CIA severity (Fig. 7A). First, the effects of AAV-as-miR-548e on miR-548e levels in synovial fibroblasts were confirmed (Fig. 7B). We found that expression of as-miR-548e alone, without the need for MSC transplantation, mimicked the therapeutic effects of MSCs on CIA, based on analyses of paw thickness (Fig. 7C), clinical arthritis score (Fig. 7D) and histological arthritis score (Fig. 7E). Together, these data suggest that MSC transplantation may alleviate experimental RA through suppressing miR-548e-mediated IκB inhibition.

## Discussion

Several studies have reported the therapeutic effects of allogeneic or xenogenic MSC transplantation in CIA-mice^30^,^31^,^32^,^33^,^34^,^35^,^36^,^40^,^41^. Nevertheless, the underlying mechanisms are poorly understood. So far, most emphasis has been placed on studying the immunoregulatory effects of MSCs. No studies have reported the regulation of NF-κB by MSCs. Indeed, NF-κB is known to be a key player in the pathogenesis of RA, and is central to the production of proinflammatory mediators in the inflamed synovium. However, the molecular regulation of NF-κB signaling is largely unknown.

In this study, we first confirmed the therapeutic effects of MSCs on CIA mice, using generally applied quantification methods, including the paw thickness, clinical arthritis score, histological arthritis score, and modulation of proinflammatory cytokines, like IL-1β, IL-6, TNF-α, IL-10 and TGFβ1. Among these cytokines, IL-1β, IL-6 and TNF-α are known to promote immune responses in RA, while the effects of IL-10 and TGFβ1 on immune responses in RA are generally believed to be negative. The effects of MSCs on these cytokines support a reduced immune reaction by MSC transplantation.

Next, we examined the effects of MSCs on NF-κB activity in this model. We analyzed the levels of the major component of NF-κB, p65, as well as the key inhibitor IκB, in synovial fibroblasts. We found that MSC transplantation significantly decreased the nuclear levels of p65 in synovial fibroblasts, suggesting that NF-κB activity in synovial fibroblasts is significantly reduced by MSC transplantation. Since NF-κB activities are regulated by many factors, and IκB is central among these factors, we examined levels of IκB. Our finding of protein but not mRNA changes in IκB by MSCs led to the hypothesis that MSCs may modulate IκB through post-transcriptional control.

Since miRNAs play a key role in the translational control of genes, we screened IκB targeting miRNAs, and specifically found that miR-548e levels in synovial fibroblasts were altered by MSC transplantation. The levels of other IκB targeting miRNAs were not significantly changed by MSCs. Using synovial fibroblasts, we found that miR-548e targets the 3′-UTR of IκB mRNA to inhibit its translation. Moreover, by challenging isolated synovial fibroblasts with TGFβ1 with or without its receptor inhibitor^42^, we found that TGFβ1 alone mimicked the effects of MSCs on the changes observed in miR-548e and NF-κB/IκB levels in synovial fibroblasts. These data suggest a model in which MSCs produce and secrete TGFβ1, which activates the TGFβ receptor in synovial fibroblasts^43^, leading to the suppression of miR-548e and, subsequently, increases in IκB levels and decreases in NF-κB levels. This model may be further confirmed in the future using fibroblast-specific inducible TGFβ receptor knockout mice.

Although synovial fibroblasts appear to be the major target of MSCs, MSCs may also regulate the proliferation and differentiation of lymphocytes, e.g. T regular cells. These aspects may be further analyzed in the future approaches.

Finally, we performed another two *in vivo* experiments to confirm the importance of miR-548e to the therapeutic effects of MSCs on CIA-mice. First, expression of miR-548e in the joints of MSC-grafted CIA-mice, which antagonized the suppression of miR-548e in synovial fibroblasts by MSCs, abolished all the therapeutic effects of MSCs on CIA severity. Second, expression of as-miR-548e alone in CIA-mice, which decreased miR-548e in synovial fibroblasts, mimicked the effects of MSC transplantation without the need for MSCs. These data strongly suggest that MSC transplantation may alleviate experimental RA at least partially through suppressing miR-548e-mediated IκB inhibition. In the current study, we used AAV, rather than adenovirus to mediate the miRNAs, which should have very limited effects on the host immune system^44^,^45^,^46^,^47^.

To the best of our knowledge, this is the first study to show that the therapeutic effects of MSCs on CIA-mice are partially mediated through miRNA-regulated NF-κB signaling suppression. Recently, Liu *et al*. showed that miR-937 inhibited translation of Brn-4 mRNA through binding to the 3′-UTR of the Brn-4 mRNA in MSCs. Moreover, transplantation of as-miR-937-expressing MSCs significantly reduced the deposition of Abeta, increased the levels of BDNF, and significantly improved the appearance of mice in an Alzheimer’s Disease model^48^. Analagously, we modified MSCs with microRNAs to improve their therapeutic effects in an RA model.

## Additional Information

**How to cite this article**: Yan, X. *et al*. Mesenchymal stem cells alleviate experimental rheumatoid arthritis through microRNA-regulated IκB expression. *Sci. Rep.* **6**, 28915; doi: 10.1038/srep28915 (2016).
